# VCF observer: a user-friendly software tool for preliminary VCF file analysis and comparison

**DOI:** 10.1186/s12859-024-05860-0

**Published:** 2024-09-03

**Authors:** Abdullah Asım Emül, Mehmet Arif Ergün, Rumeysa Aslıhan Ertürk, Ömer Çinal, Mehmet Baysan

**Affiliations:** 1 Department of Computer Engineering, Istanbul Technical University, Istanbul, Turkey; 2 Health Institutes of Türkiye, Istanbul, Turkey

**Keywords:** VCF, Comparison, Benchmarking, Visualization, Graphical, User-friendly

## Abstract

**Background:**

Advancements over the past decade in DNA sequencing technology and computing power have created the potential to revolutionize medicine. There has been a marked increase in genetic data available, allowing for the advancement of areas such as personalized medicine. A crucial type of data in this context is genetic variant data which is stored in variant call format (VCF) files. However, the rapid growth in genomics has presented challenges in analyzing and comparing VCF files.

**Results:**

In response to the limitations of existing tools, this paper introduces a novel web application that provides a user-friendly solution for VCF file analyses and comparisons. The software tool enables researchers and clinicians to perform high-level analysis with ease and enhances productivity. The application’s interface allows users to conveniently upload, analyze, and visualize their VCF files using simple drag-and-drop and point-and-click operations. Essential visualizations such as Venn diagrams, clustergrams, and precision–recall plots are provided to users. A key feature of the application is its support for metadata-based file grouping, accomplished through flexible data matrix uploads, streamlining organization and analysis of user-defined categories. Additionally, the application facilitates standardized benchmarking of VCF files by integrating user-provided ground truth regions and variant lists.

**Conclusions:**

By providing a user-friendly interface and supporting essential visualizations, this software enhances the accessibility of VCF file analysis and assists researchers and clinicians in their scientific inquiries.

## Background

Advancements in DNA sequencing technology and computational power have the potential to transform the landscape of medicine, allowing for personalized treatments based on patients' genetic information. Variant call format (VCF) is the primary storage format for genetic variant data, playing a fundamental role in genomic analyses. VCF files store sequencing data based on how a given sequence deviates from a reference sequence. Each point of deviation is described by the chromosome and position within that chromosome it occurred on, the base(s) found at that position in the reference, and the corresponding base(s) in the given sequence. This is a great strength of the format as storing entire sequences is not needed given the high degree of similarity between different genomes. Still, despite this greater optimality, VCF files frequently contain thousands to millions of variants. This presents a challenge to researchers seeking insights into the diverse set of VCF files they are working with.

One of the main questions faced when obtaining VCF files from sequencing data is which tools to use for the various steps involved in that process. These steps contain, for example, aligning the given sequence to the reference sequence and determining if a deviation from the reference at a certain position indicates a variant at that position. The choice of which set of tools (commonly called pipelines) to use can determine whether an analysis provides meaningful and actionable results. Due to the varying strengths of different tools, in many cases it is worthwhile to run analyses using different pipelines and pick variants based on the outputs of a combination of them providing the best results given the issue at hand [1–3]. This requires the comparison and benchmarking of the VCF files produced by the various pipelines.

Editing, comparing, and visualizing VCF files are frequently done tasks in genomics. The availability of software tools with graphical user interfaces (GUIs) is crucial in broadening the usefulness of the high volumes of data made available to researchers [4–6]. More specifically, enabling individuals lacking programming experience to view, analyze, and understand the data they have is a crucial step in expanding the utilization of genomics in medical/clinical settings. Genomics data has much unrealized potential to be a catalyst for advancements in personalized medicine. Taking advantage of the full potential of genomics is critical for the transition of healthcare practice toward a paradigm of treatment tailored for individuals. By using individuals’ genetic makeups, healthcare systems can provide personalized treatments and enhance diagnostic accuracy and treatment effectiveness.

There currently exist many tools to address the various challenges in VCF file handling. Table 1 summarizes major tools’ capabilities and compares them with VCF Observer, the tool we are presenting in this paper. VCFtools, a command line application, is the most fundamental tool available which operates on VCF files. It is a tool that was developed alongside the variant call format and facilitates many basic operations commonly performed on VCF files, such as filtering and comparison. It does not, however, provide visualizations and is not available outside of the command line [7]. BCFtools is another major command line utility that promises greater performance than VCFtools but requires prior compression and indexing of VCF files before performing operations such as comparison on them. BCFtools also does not have a graphical interface, does not provide visualizations, and does not offer support for grouping or benchmarking VCF files [8]. VCFtools and BCFtools do not offer distinct benchmarking capabilities, however, they can be used to put together benchmarking results based on their comparisons.

**Table 1:** Summary of VCF file handling tools and their capabilities

	Comparison	GUI	Filtering	Benchmarking	Visualization	Metadata
VCFtools	×		×			
BCFtools	×		×			
BrowseVCF		×	×			
VCF-miner		×	×			
VIVA			×		×	×
123VCF		×	×			
VCF observer	×	×	×	×	×	×

Features present in various tools for handling VCF files are marked with a cross. Only command line tools allow the comparison of VCF files while they do not provide visualization capabilities. Filtering capabilities offered by VCF-Miner, BrowseVCF, and 123VCF allow the use of custom annotations while the others do not

VCF-Miner [9], BrowseVCF [10], and 123VCF [11] are tools that allow users to filter VCF files dynamically according to the annotations present in them and by genomic regions. They provide graphical interfaces and allow users to concentrate on the subset of variants they are interested in. BrowseVCF allows exporting the filtering steps that have been used for a given query, to provide reproducibility for the filtering, while 123VCF performs filtering based on a user-specified file for the same purpose. These tools, however, are not capable of comparisons between different files and do not offer visualizations.

VIVA is a software tool that is available both on the command line and as a Julia package [12]. It was developed to simplify the visualization process of VCF files. It can filter a VCF file and produce visualizations. It accepts metadata related to samples present in a VCF file, allowing the metadata’s use for sorting and filtering. It provides heatmaps and scatter plots which can express genotype and read depth information for samples and variants. It also offers multiple file formats in which to export the figures it produces. VIVA does not offer a graphical interface to users. It does not compare VCF files with one another and does not provide benchmarking capabilities.

We have developed VCF Observer, a VCF file analysis and comparison web tool, to address these issues. It can calculate similarity between VCF files and benchmark them based on user-provided validation sets. It supports the dynamic grouping of multiple VCF files based on user supplied metadata, facilitating the interpretation of relations between different sets of VCF files. It can also filter VCF files based on genomic regions and the filter status of variants. Results are provided in the form of visualizations, CSV (comma separated values) files, and VCF files.

The primary focus of this software tool is enabling researchers to conveniently perform basic analyses and comparisons, which are often cumbersome using existing tools. Unlike many current VCF file analysis methods that lack graphical interfaces, VCF Observer offers a seamless and intuitive user experience. Researchers can upload their VCF files to the web interface and efficiently analyze them, using the most commonly preferred visualizations in bioinformatics: Venn diagrams, clustergrams, and precision–recall plots. They can also download the results of their analyses in the form of images and variant lists.

VCF Observer facilitates VCF file metadata integration via CSV files. A data matrix containing each VCF file being analyzed and a user-chosen number and variety of properties for each file is accepted to dynamically group VCF files prior to analysis. This can be used, for example, to group VCF files based on which tools produced them, thus facilitating the exploration of how functionally equivalent tools compare to one another.

VCF Observer was developed using the Python 3 programming language and the Dash library for web application development. It was designed to have a user-friendly and uncluttered interface and can be used both by those unfamiliar with bioinformatics and by more experienced users. By offering filtering and analysis of variant data, widely used visualizations, and metadata-driven file grouping, it enables researchers and clinicians to perform quick, high-level analyses on VCF files.

### Implementation

The main functionality of VCF Observer is the analysis, comparison, and visualization of VCF files. It was developed using the Python (3.10.8) programming language and makes extensive use of the Dash (2.7.1) library for web development. Dash provides a framework for creating interactive data visualization web applications based on Flask and React.js. We used the Dash Bootstrap Components (1.2.1) library to design the application’s layout and the Dash Bootstrap Templates (1.0.7) library for styling. VCF files are loaded by the application via the scikit-allel library and stored in Pandas data frames. Visualizations are produced using the common Python data visualization libraries Matplotlib (3.6.2), venn (0.1.3), Plotly (5.11.0), and Dash Bio (1.0.2). VCF Observer can be run on any platform that supports Python 3.8 or later, such as Windows 10. Standalone versions of the application (created using cx_Freeze) that do not require Python to be installed on the system are also provided for Windows, MacOS, and Linux operating systems. The source code and standalone releases of the application are available on GitHub. The application can also be reached via https://bioinformatics.itu.edu.tr/vcf-observer. User data uploaded to this address is temporarily stored for 24 h after which i̇t is deleted. When the application is run locally, no data leaves the user’s device.

The Dash framework is structured such that a layout definition for the app is specified then “callback” functions define the interactive behavior of the application by describing how the layout will update according to the user’s actions. This means that software developed using Dash follows the model–view–controller (MVC) software design pattern. The model is the data being used and visualizations being produced, the view is the layout, and the controller is the set of callback functions.

VCF Observer works in two stages: loading data and performing analysis. When data is uploaded by the user i̇t is processed into data frames and cached by the server. All file uploads accept multiple files. VCF files compressed with GZip or Zip are also accepted. If any errors are encountered, they are presented to the user and the data in question is not cached. Due to technical limitations, there is a size limit of 200 MB per file. Three file formats are accepted: variant call format (VCF), comma separated values (CSV), and browser extensible data (BED). Uploaded VCF files are categorized into two groups: “compare set” and “golden set”. The compare set contains files that are to be analyzed, compared, and visualized while the golden set is used to calculate the precision and recall values of the compare set for benchmarking. VCF files describe variants according to the variants’ chromosome, position, reference, and alternative. When loading variant data, VCF Observer uses this information to create an ID for each variant. Filter column information is also loaded to allow for the filtering of variants when performing analysis.

CSV files are used to store data that is in tabular form. VCF Observer supports the use of CSV files to describe the properties of each VCF file in the compare set. This data matrix is expected to contain a column labeled “FILENAME” and list each file in the compare set under this column. It can have as many other columns as desired by the user, describing the properties of the files in the compare set. These properties can then be used to dynamically group files and juxtapose data of differing origin, for example. BED files contain information about genomic regions. They can be used to describe portions of interest in a genome and allow researchers to filter genomic information, such as variant lists. Our application provides VCF file filtering according to genomic regions provided by the user, as well as regions offered by the application. Genomic regions offered by the application can be configured on the server side by placing BED files in the application directory. Filtering according to variant type (SNP/indel) and chromosome number is also provided.

When VCF Observer receives an analysis request, and the files necessary to fulfill this request have been successfully loaded, i̇t first performs filtering on the compare set by keeping or removing variants based on their filter column information and then based on the selected genomic regions as well as chromosome and variant type. Then, if metadata was provided and columns by which to group VCF files were selected, files in the compare set are grouped according to their metadata such that the variants they hold are pooled. There are three methods offered for combining files: union (variants are included if they are present in any file in the group), intersection (variants are included only if they are present in all files in the group), and majority (variants are included only if they are present in > 50% of files in the group). These groups are used as the basis of analysis similarly to files. When labeling groups, a subset of the properties of each group can be returned based on user selection. This is useful when the user is interested in a specific property of the groups.

VCF Observer offers four analysis types: tabulated variant counts, Venn diagrams, clustergrams, and precision–recall plots. Tabulated variant counts contain listings of the number of variants in each file in the compare set. If metadata is used to generate groups, pivoting functionality is also provided where each axis of the table contains different group properties.

**Fig. 1:**
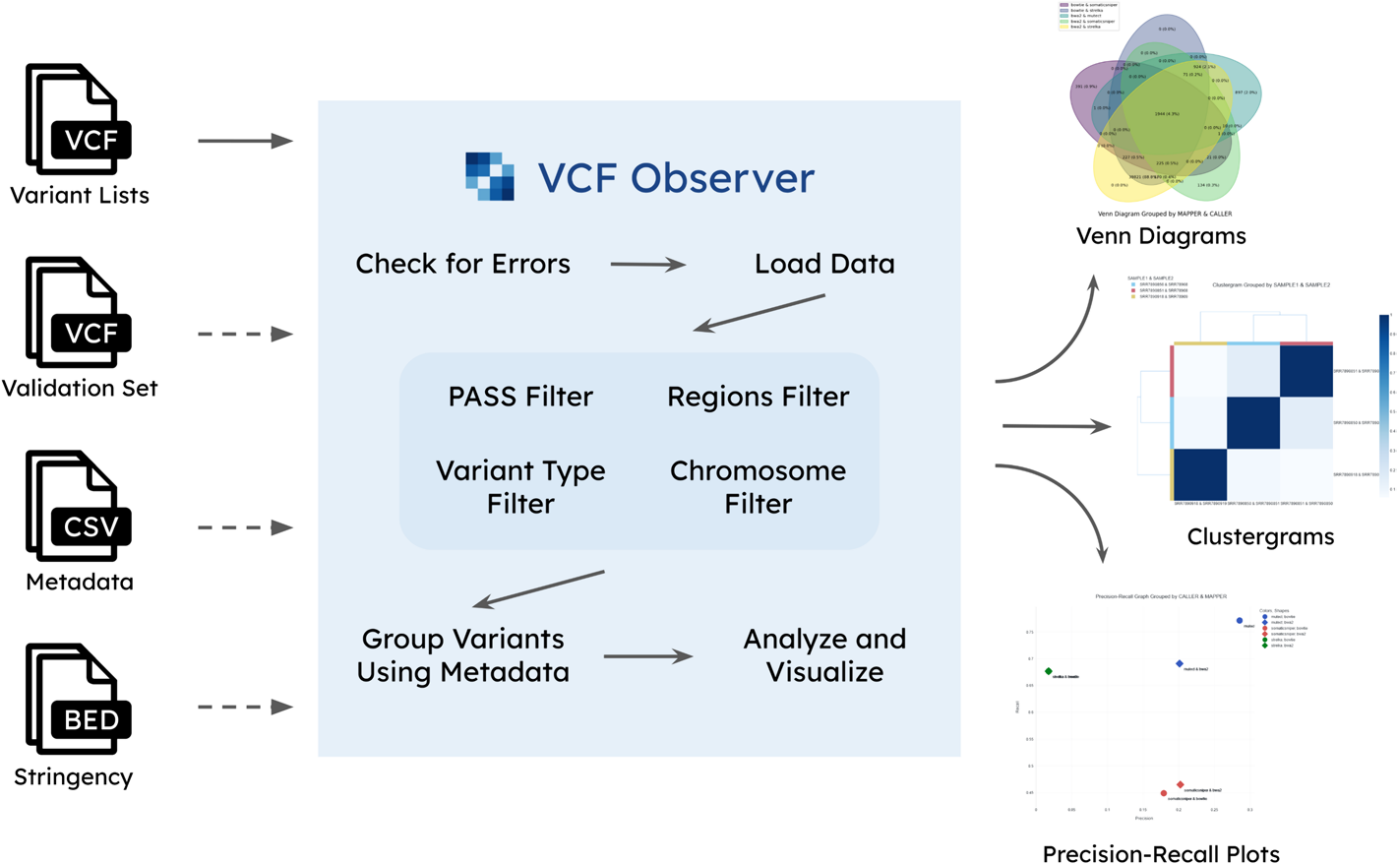
Overview of the inputs, outputs, and internal workflow of VCF Observer. VCF files for comparison are required inputs while validation sets (also VCF), metadata (CSV), and stringency (BED) are optional. Workflow steps include checking for errors, loading data, applying PASS filtering, applying genomic regions filtering, grouping variants using metadata, and analysis and visualization. If errors are detected, they are returned to the user, instead of the following steps being executed. Venn diagrams, clustergrams, and precision–recall plots can be generated

Venn diagrams are used to visualize the degree of overlap between the variants present in files. Up to 6 sets can be visualized in this way. An option to generate a "pseudo-Venn diagram" that provides more readability for cases with 6 sets is also provided. Clustergrams (heatmaps with dendrograms showing clusters of rows and columns) are used to compare files based on their Jaccard distance (the number of variants in their intersection divided by the number of variants in their union). Precision–recall plots provide benchmarking capabilities based on the uploaded golden set. Data points can be customized to reflect metadata. For example, data point colors may be based on one column and shapes based on another, providing a way for the user to view performance variations in different categories on the same plot. Figure 1 summarizes VCF Observer’s operation.

Precision is calculated as the ratio of the number of correct variants in a file (or grouping) from the compare set to the number of variants in that file. Recall is calculated as the ratio of the number of correct variants in a file (or grouping) from the compare set to the number of variants in the golden set. The correct variants are defined as those in the intersection of a file (or grouping) in the compare set with the golden set. A variant is taken to be in the intersection of two sets if there is an exact match in both sets. If the set of variants in a compare set file (or grouping) is C and the set of variants in the golden set is G, the following equations give precision and recall:

$$Precision = \frac{{\left| {C \cap G} \right|}}{\left| C \right|} \quad\quad Recall = \frac{{\left| {C \cap G} \right|}}{\left| G \right|}$$

Filtering based on genomic regions is done using an algorithm we developed that we implemented in Python. Variant positions are kept or removed based on whether they fall within regions specified in the BED files chosen by the user. In our initial design, the algorithm checked every possible region for each variant, yielding an algorithmic complexity of O(n^2^). We noticed that, because we sorted genomic regions and variant lists before caching them, we could ignore checking later regions for a variant if the variant had a position falling after a given region. This meant that each set (variants and regions) only needed to be iterated over once. Thus, in cases where the assumption of sortedness can be made, this type of optimization allows for a time complexity of O(n). Test results comparing our initial algorithm’s performance with that of the optimized version are given in Performance.

All analysis results provided by VCF Observer are made available for download. Clustergrams and precision–recall plots can be downloaded using the download option in their interactive windows while Venn diagrams are provided with a separate download button. All images are made available as PNG files. Text-based results are provided as CSV files while variant intersection sites are made available as compressed VCF files.

## Results and discussion

We developed VCF Observer, a graphical web tool that can analyze, compare, benchmark, and visualize VCF files. Although there are various tools for comparing VCF files, none provide a graphical interface or visualizations. Our software tool provides this functionality and makes working on VCF files more accessible.

### User interface

VCF Observer’s user interface is separated into two parts: a navigation bar (navbar) is present on the left side of the screen and a display area covers the rest. Maintaining visual consistency has been a key consideration throughout the development process to ensure an intuitive and user-friendly experience. The navbar provides users a way to navigate the website and the display area presents the results of user actions, such as uploading files and requesting analyses. There are three tabs available in the navbar: Welcome, Upload, and Analyze. Each updates the display area with its information when selected. When first visiting the website, users are greeted with the Welcome tab that describes the functionality of the website and its overall layout. They can use the button at the bottom of the navbar to continue to the Upload tab. There, they can upload files they are interested in working with and move on to the Analyze tab. Lastly, they can select an analysis, request i̇t, and view its result. On subsequent visits, users are automatically navigated to the upload tab.

**Fig. 2:**
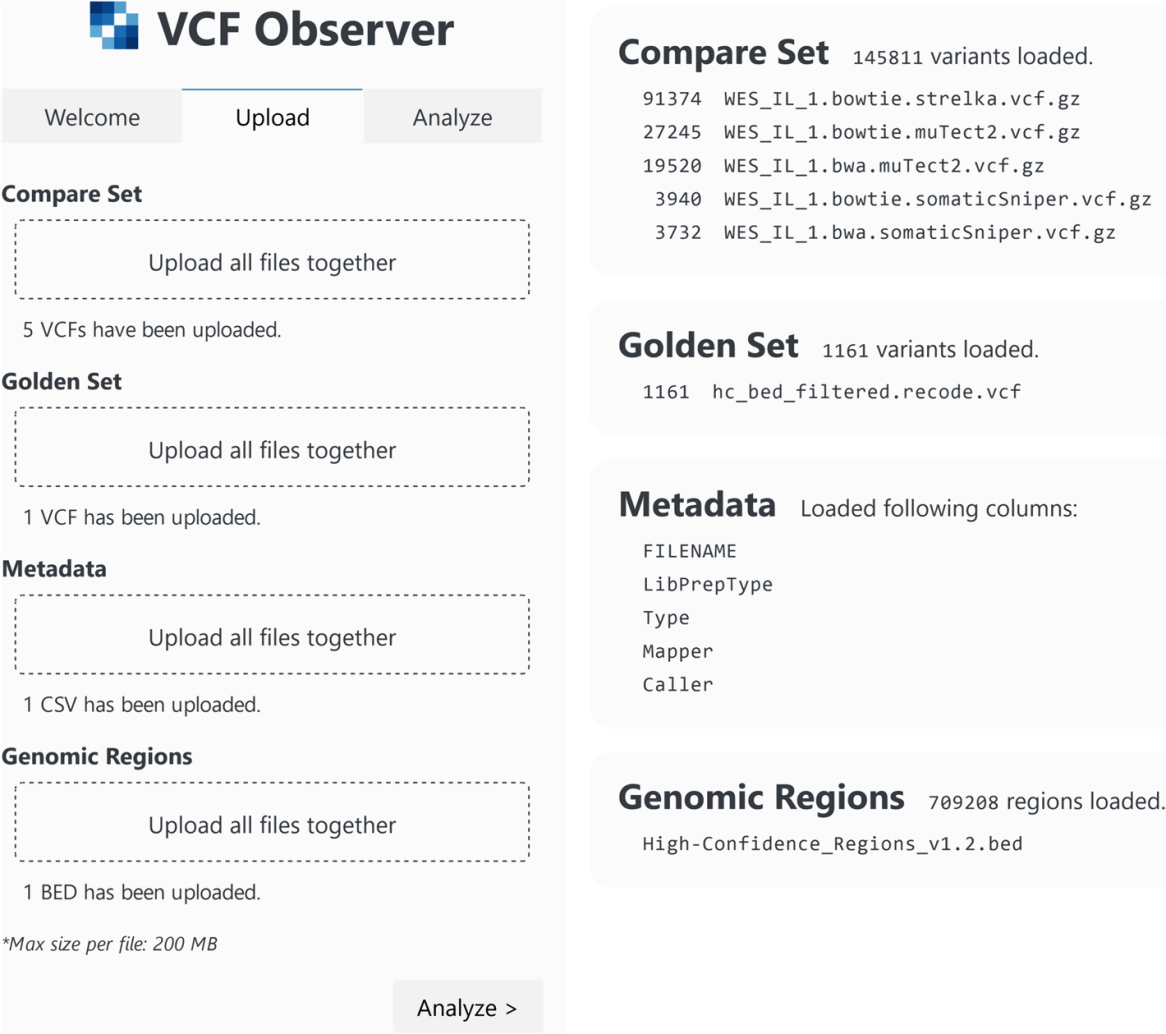
The Upload tab of VCF Observer. Successful upload results are shown. The upload status of files can be seen under each upload box in the navbar on the left. The successful upload results for all categories of files can be seen in the display area to the right

The Upload tab contains 4 upload boxes for the file categories accepted by the application. These are the compare set, golden set, metadata, and genomic regions. The display area shows 4 upload result summary cards corresponding to the 4 file categories. Files can be dragged-and-dropped onto the upload boxes or the boxes may be clicked to open the browser’s file selection dialog for the upload of files. If uploads are not successful, users are notified with a message below the upload box and details of the problem are shown in the display area. Upon successfully loading a category of files, the number of files loaded in that category are shown below the upload box. When the compare set or the golden set is successfully loaded, the number of variants present in each file that has been uploaded is shown in their respective upload result summary cards, listed according to each file’s number of variants in descending order. VCF files describe variants according to the variants’ location in the genome and the change that was detected there. VCF Observer assigns IDs to variants in each VCF file using this information for use during analysis. Upon successful loading of metadata, the columns describing the compare set present in the metadata are shown in the display area. For genomic regions, the display area lists the filenames of uploaded files and only shows the aggregate count of the number of regions loaded. The Upload tab and its display area showing the results of uploads for all categories can be seen in Fig. 2.

The Analyze tab has a radio selector containing the 4 analysis types offered by VCF Observer: Data Summary, Venn Diagram, Clustergram, and Precision–Recall Plot. Data Summary offers 3 views: variant counts per file, variant counts based on compare set grouping, and listings of loaded data. Variant counts per file in either the compare set or golden set can be viewed as a histogram or as a table. Variant counts based on compare set grouping are generated using the metadata of files in the compare set. Files are grouped together based on dynamically defined properties in the metadata. There are three methods provided when grouping files: union, intersection, and majority (see Implementation for details). This style of grouping is also available for the Venn diagram, clustergram, and precision–recall plot analyses. In this view, there is also the option to pivot the table such that some metadata columns are present along the x-axis rather than the y-axis. The last data summary view provides a raw listing of the data loaded into VCF Observer in the form of tables.

**Fig. 3:**
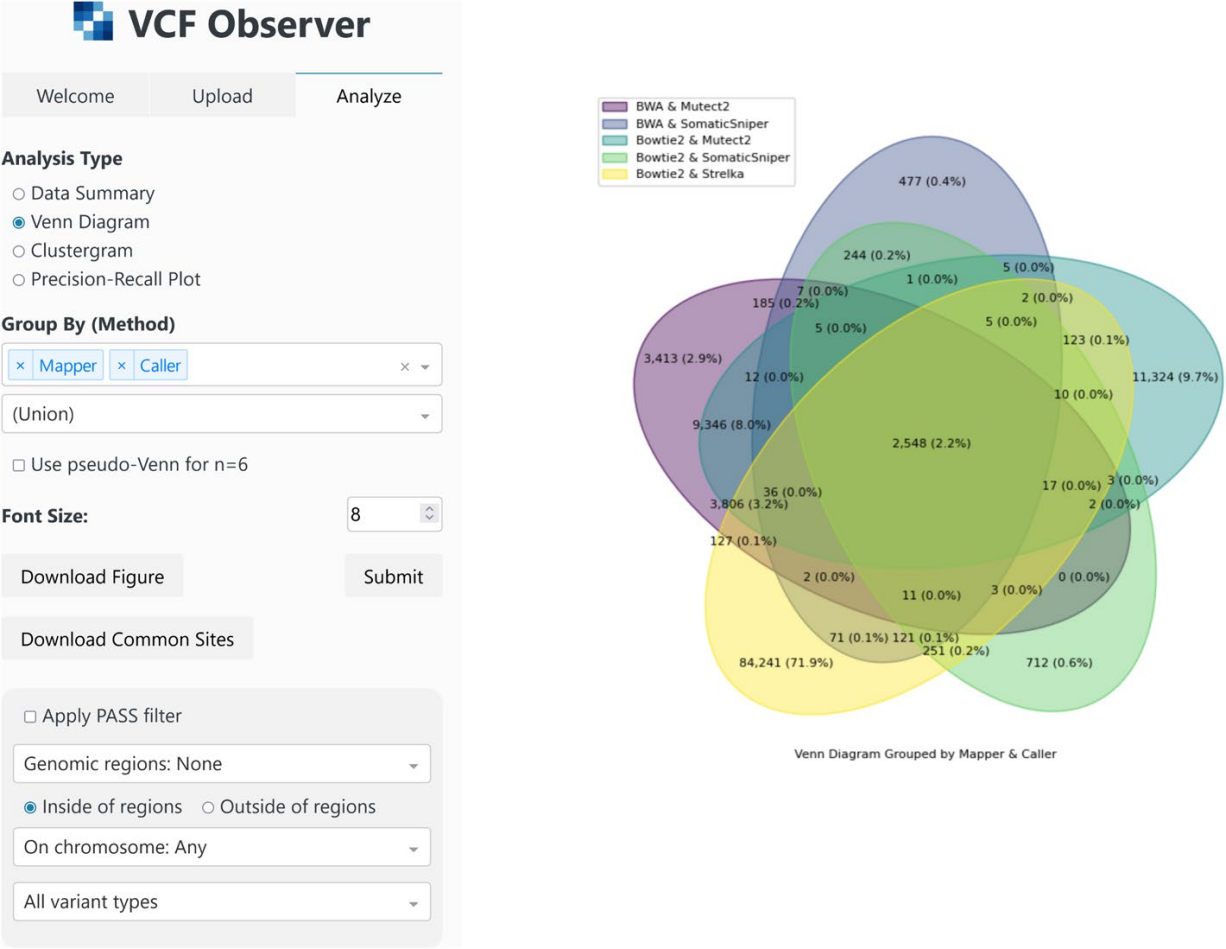
The Analyze interface of VCF Observer. A Venn diagram generated with the settings presented in the navbar on the left is shown. The numbers of variants in each intersection of 5 sets is shown along with the percentages these represent in parentheses

Venn diagrams are available for visualizing up to 6 sets (files or groups of files). There is an option to generate a pseudo-Venn diagram for cases with 6 sets to aid in visual clarity. The variants in the intersection of all sets are provided for download as well as the figure image. Clustergrams (heatmaps with dendrograms visualizing the similarity of rows and columns) are provided to visualize similarity among files in the compare set using their Jaccard distance. Both axes of the heatmap contain all files to be compared and comparisons are shown for each file pair. The labels shown for files can be determined based on metadata such that any combination of metadata columns may be used for labeling. Rows and columns can also be color-coded based on their labels to increase the readability of the clustergram. Various coloring schemes for the heatmap are also provided to the user. Precision–recall plots are provided for the purpose of benchmarking. For each file being benchmarked, precision is shown on the x-axis and recall on the y-axis in the form of a scatter plot. Labels can be chosen for each file in the same way as described for clustergrams. Additionally, the shapes and colors of data points on the plot can be set according to values in one or more metadata columns, allowing for patterns resulting from differences in file properties to be more easily visible. All visualizations are provided with the option of setting the font size, allowing for effective use in various styles of presentation. Lastly, the bottom of the navbar Analyze tab contains options for filtering variants prior to analysis. VCF files contain a FILTER column describing whether each variant passed the filters imposed by the calling algorithm that found i̇t. Variants that have passed such filtering are marked with a value of PASS in their filter columns. In VCF Observer, variants that have a FILTER column value of PASS can be selected for analysis exclusively. Also, variants can be filtered according to whether they fall inside or outside of certain genomic regions. Genomic regions can be provided by the user or by the server. Filtering options based on variant type and chromosome number are also present. The Analyze tab and its display area showing a generated Venn diagram can be seen in Fig. 3.

For all analysis types, upon successful completion of the analysis, an option to download resultant data is provided. For clustergrams and precision–recall plots, the download option is given in the figures’ interactive windows. Images are provided as PNG files and text-based results are provided as CSV files. Variant intersection sites computed as part of the Venn diagram are provided as compressed VCF files.

### Use cases

VCF Observer’s main utility is comparing and benchmarking VCF files and visualizing the results of these operations. To this end, it provides additional capabilities such as filtering and grouping. It can be used, for example, to determine which set of tools is best suited to call variants from a given set of sequence reads, by benchmarking the results produced by each candidate. As another example, given a set of whole genome variant lists derived from various samples, it can be used to filter out intron variants and show the degree of similarity between the variants in the exomes of these samples using a clustergram. To concretely demonstrate these capabilities, we used two pre-existing datasets provided by the SEQC2 consortium: WGS-based (whole genome sequencing) germline variant call sets and WES-based (whole exome sequencing) somatic variant call sets [13]. We chose these datasets due to their inclusion of VCF files produced using various different tools (differing aligners and callers in both cases) but using the same sequence reads. Comparing such files was our primary motivation during development.

**Fig. 4:**
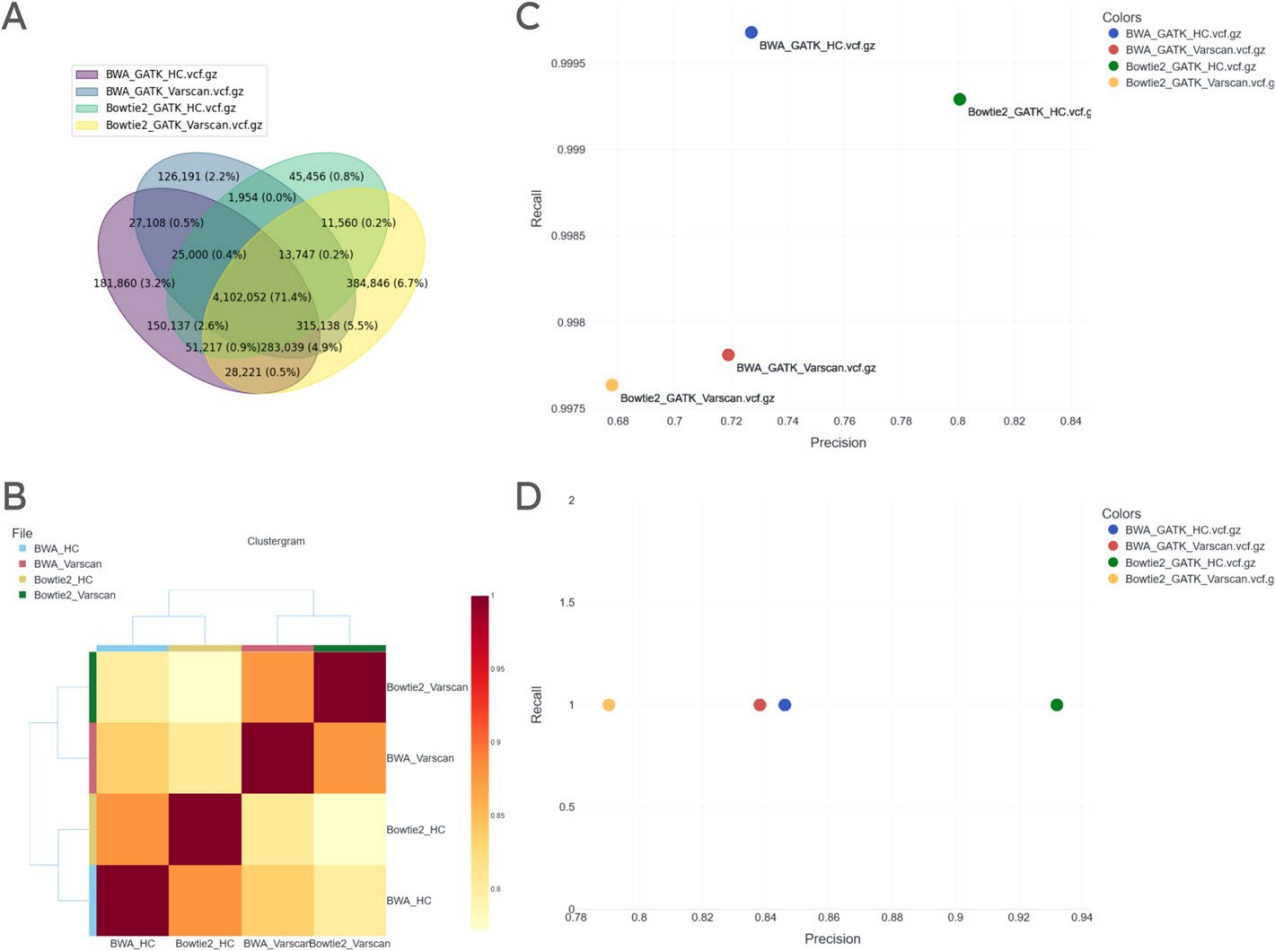
Visualizations generated using 4 VCF files from the SEQC2 consortium’s germline WGS analysis of NA10835. **A** Venn diagram comparing variants in VCF files. **B** Clustergram showing pairwise Jaccard distances of VCF files. **C** Precision–recall plot calculated based on highly reproducible regions created by the SEQC2 consortium. **D** Precision–recall plot where the golden set is the intersection of all 4 VCF files

From the germline calling dataset we selected 4 VCF files created with two different aligners (BWA and Bowtie2) and two different callers (GATK’s HaplotypeCaller and GATK’s VarScan2). We chose files that had GRCh38 as their reference and that were marked as being derived from well A01. In order to observe how these files differed from one another, we produced a Venn diagram (Fig. 4A). Here, we saw that, of the ~ 5.7 million unique variants (~ 19 million including duplicates), ~ 4.1 million were present in all 4 files, corresponding to ~ 71.4% of all unique variants. The two BWA files shared a distinct ~ 5.8% between them while the Bowtie2 files shared a distinct ~ 1.3%. The VarScan2 files shared another ~ 10.6% in addition to the prior ~ 71.4% and the HaplotypeCaller files shared another ~ 3.9%. We concluded that the VCF files produced from BWA alignments were more similar amongst themselves than those from Bowtie2 alignments. Similarly, the VCF files generated by VarScan2 were more similar than those generated by HaplotypeCaller. To obtain a simpler overview of similarity information, we generated a clustergram (Fig. 4B). Here we saw that the lowest similarity score (Jaccard distance) was ~ 0.75, loosely mirroring the ~ 71.4% shared variants amongst all files. Jaccard distance is calculated pairwise for each case and thus is not directly related to overall similarity, although we can observe loose correlation in this case due to the latter’s high value. Files were clustered by their callers, while aligners appeared to have a less significant effect on similarity. Lastly, we wanted to benchmark these VCF files. We chose the highly reproducible regions provided by the SEQC2 consortium [14] as a golden set and produced a precision–recall plot (Fig. 4C). Recall values for all 4 VCF files were > 0.99, with the VCF file generated using BWA and HaplotypeCaller having the highest value. Precision values, on the other hand, showed greater variation in the range of 0.67–0.81, with VarScan2’s results being worse than HaplotypeCaller’s. To demonstrate the variant intersection sites selection of VCF Observer, we downloaded the intersection variant set provided via the above-described Venn diagram analysis and uploaded it as a golden set. We generated a precision–recall plot using this new golden set (Fig. 4D). All recall values were 1.0, because the golden set in this case was a subset for all files. We observed that the VCF file obtained using Bowtie2 and HaplotypeCaller was the most similar to the intersection set of all four files, while the file obtained using Bowtie2 and VarScan2 was the least similar.

**Fig. 5:**
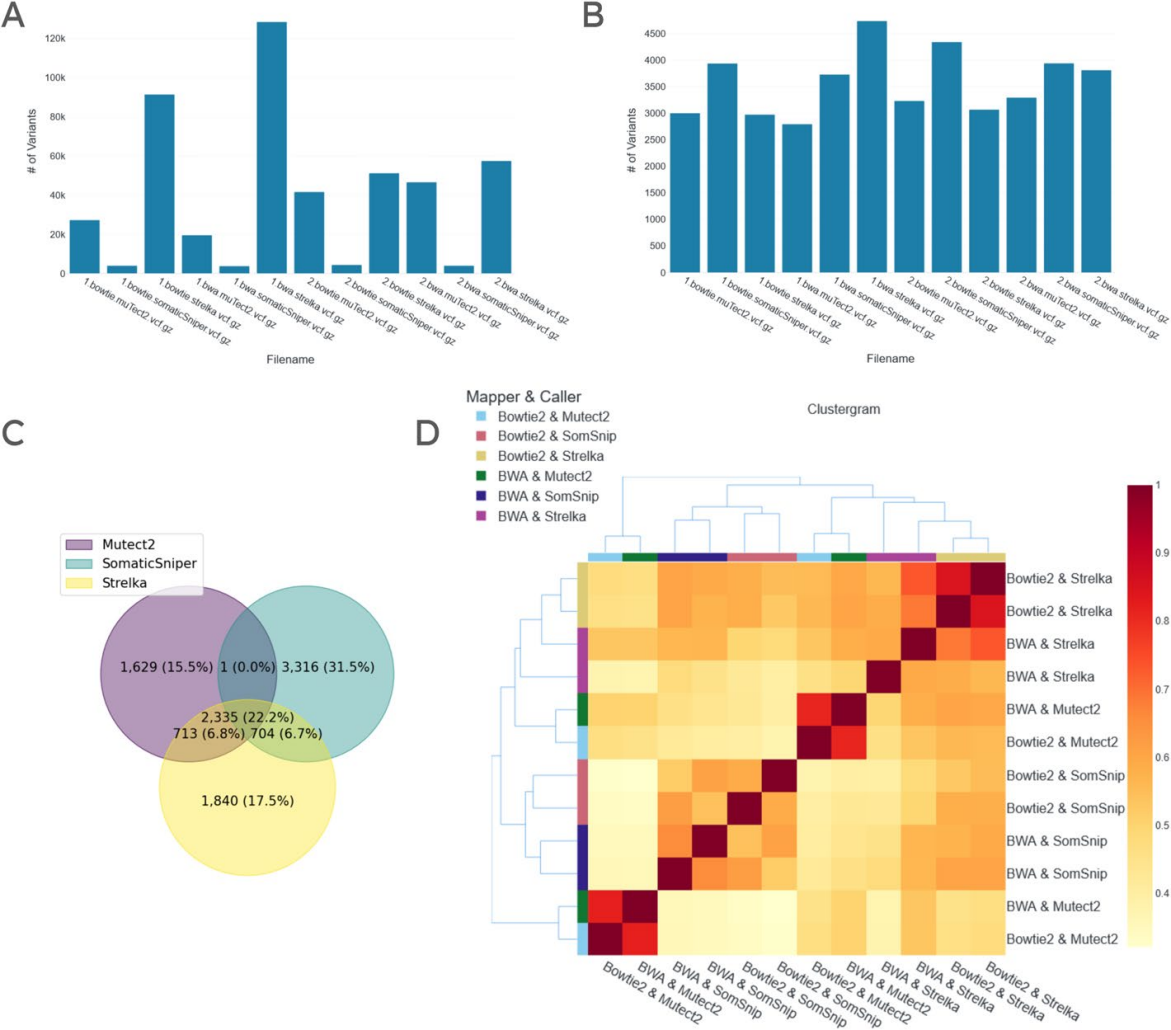
Visualizations generated using 12 VCF files from the SEQC2 consortium’s somatic WES analysis of HCC1395BL (normal) and HCC1395 (tumor). **B**–**D** were produced after the 12 VCF files were PASS filtered. **A** Histogram of variant counts for each file with no preprocessing applied. **B** Histogram of variant counts for each file with PASS filter applied. **C** Venn diagram comparing variants, generated after files produced by the same callers were grouped via union. **D** Clustergram showing pairwise Jaccard distances of VCF files. SomSnip: SomaticSniper

**Fig. 6:**
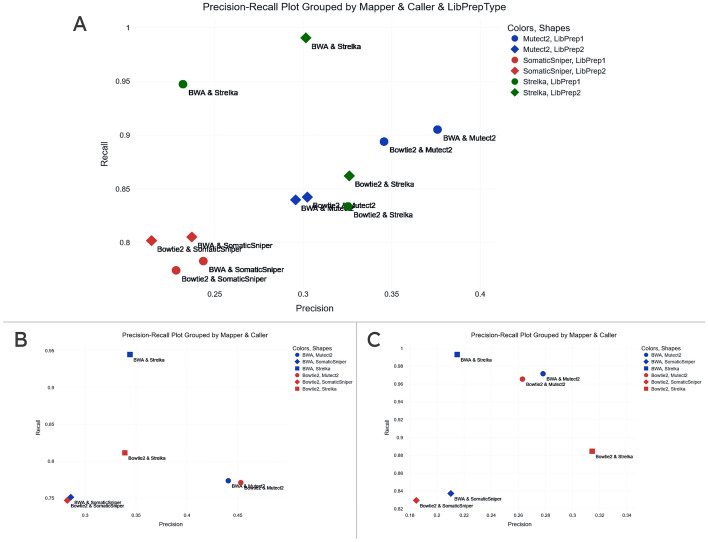
Precision–recall plots generated using 12 VCF files from the SEQC2 consortium’s somatic WES analysis of HCC1395BL (normal) and HCC1395 (tumor) as well as high-confidence regions created by the SEQC2 consortium as the golden set. **A** Scatter plot showing benchmarking results for all 12 VCF files. **B** Scatter plot showing benchmarking results for the intersections of VCF files sharing the same aligner and caller. **C** Plot showing benchmarking results for the unions of VCF files sharing the same aligner and caller

From the somatic calling dataset we selected 12 VCF files created using 2 different aligners (BWA and Bowtie2), 3 different callers (Mutect2, SomaticSniper, and Strelka), and 2 different library preparation methods. To get an overview of the VCF files, we generated a histogram showing variant counts (Fig. 5A). Here we noticed that some VCF files produced by Strelka had significantly more variants (on the order of 100,000) while those produced by SomaticSniper had significantly fewer (on the order of 1000). The rest of the files had variant counts on the order 10,000. This indicated a possibility that some of these files had been PASS-filtered by their respective callers while others had not. For this reason, we applied a PASS filter on all files and generated a new histogram (Fig. 5B). In this visualization we saw that all VCF files had variant counts on the order of 1000, confirming our prior conjecture. We performed all subsequent analysis with the PASS filter option enabled. We created a CSV file containing the aligner, caller, and library preparation associated with each file, so that we could group and label them dynamically. We produced a Venn diagram after grouping the files with a union operation using their callers (Fig. 5C). We saw that, of all unique variants, ~ 22.2% were common to all three callers’ groups. Strelka had variants in common with Mutect2 and SomaticSniper at ~ 6.8% and ~ 6.7% respectively. We also observed that SomaticSniper had twice as many uniquely identified variants compared with the other two callers. To see the effect of library preparation on the similarity between VCF files produced, we generated a clustergram where labeling excluded the library preparation so that files differing only in that aspect were marked with the same color (Fig. 5D). This showed that other than for files produced by Mutect2, the library preparation method explained the least difference between files, and the caller explained the most. In the case of Mutect2, however, there was a remarkable degree of similarity (Jaccard =  ~ 0.81, ~ 0.82) when the library preparation was the same (we regenerated the figure with library preparation type as a label to be certain of this). For Mutect2, file pairs sharing library preparation type (but differing in aligner type) were more similar to other callers than to one another (Mutect2 & LibPrep1 differed significantly from Mutect2 & LibPrep2). We benchmarked this data using high-confidence regions taken from [15]. We first produced a precision–recall plot showing values for all 12 VCF files, where data points were labeled by their aligners and callers. Data point colors showed caller type and their shapes showed library preparation type (Fig. 6A). All VCF files had recall values of > 0.85 and precision values in the range of 0.21–0.38. VCF files produced by SomaticSniper had the lowest precision and recall values. One of the two VCF files produced using BWA and Strelka had the highest recall value at ~ 0.99, while one of the two produced using BWA and Mutect2 had the highest precision value at ~ 0.37. Next, to investigate the effect of grouping pairs of files produced using the same aligner and caller combination, we generated two precision–recall plots where files differing only in the library preparation type were combined. In one plot, grouping was done using the intersection of the files (Fig. 6B) and in the other, i̇t was done using their union (Fig. 6C). In both cases, the highest recall was achieved by the variant list created using BWA and Strelka. Without grouping, the highest recall value for this combination was ~ 0.99. When grouping via union, there was a marginal increase in recall. When grouping via intersection, recall decreased to ~ 0.94. When grouping via union, the highest precision was achieved by the variant list obtained using Bowtie2 and Strelka (~ 0.31, as opposed to ~ 0.32 without grouping), in contrast to BWA and Mutect2, which produced the highest precision without grouping. This can be attributed to the decrease in the precision values of variant lists associated with Mutect2 when grouping via union. This effect, however, was not present when grouping via intersection. Mutect2’s variant lists produced using both BWA and Bowtie2 had higher precision values at ~ 0.44 and ~ 0.45 respectively, compared to values < 0.40 without grouping.

### Performance

In order to produce an overview of VCF Observer’s performance, we performed various tests of its functionality on a 2022 M2 MacBook Air with 16 GB of RAM. We ran 4 types of tests: comparing two VCF files to generate a Venn diagram, benchmarking a VCF file to produce a precision–recall plot, applying genomic regions filtering (using a BED file) to a VCF file, and applying a PASS filter to a VCF file. Each test was performed using 5 different VCF file sizes, giving a total of 20 test configurations. Each test configuration was run 10 times and their averages are presented in Table 2.

**Table 2:** Performance test results showing times (in seconds (s)) for various operations performed by VCF Observer

	1 k	10 k	100 k	1 M	10 M
Generating venn diagram (s)	0.12	0.15	0.96	9.95	120.17
Generating precision–recall plot (s)	0.05	0.13	0.86	8.6	101.43
Applying PASS filtering (s)	0.01	0.05	0.45	4.4	49.1
Applying BED filtering (s)	0.73	0.77	1.23	5.95	63.5
Applying BED filtering* (s)	0.62	0.72	1.78	12.6	137.09

Each row contains the average running time of 10 runs of a particular test performed with various input file sizes. There are five tests: “Generating Venn Diagram”, “Generating Precision–Recall Plot”, “Applying PASS Filtering”, and 2 instances of “Applying BED Filtering”. *The latter BED filtering test was performed using an earlier implementation of our genomic regions-based VCF filtering algorithm to demonstrate the effectiveness of the optimization described in Implementation. k: 1000, M: 1,000,000

The VCF file sizes used were 1000 variants, 10,000 variants, 100,000 variants, 1,000,000 variants and 10,000,000 variants. For the tests generating Venn diagrams and precision–recall plots, two VCF files were used where the files both contained the aforementioned number of variants each. In the BED filtering test, a BED file listing exome regions was used.

When working with 100,000 variants or less, VCF Observer can provide analysis results in less than 3 s (assuming an analysis consists of both filtering options and a visualization). For 1 million variants, i̇t produces results in 10–30 s. For 10 million variants results are produced in 4 min or less.

We performed genomic regions filtering tests twice: once with an unoptimized and once with an optimized algorithm (see Implementation for details). Comparing the two genomic regions filtering test results, we saw that the unoptimized version of our algorithm performed more slowly as the number of variants being processed increased. For tests with 1000 and 10,000 variants, the unoptimized algorithm had a shorter run time, whereas i̇t performed twice as slowly for tests with 1 million and 10 million variants. The theoretical time complexity calculations described in Implementation were not observable in the test results. This is because only the variant list sizes were varied while the number of genomic regions was constant.

### Future work

VCF Observer provides comparisons of VCF files and visualizes these comparisons. It offers a user-friendly graphical interface. During future development, we plan to provide more varieties of visualizations such as violin plots to show the read depths of variants in VCF files and idiograms to mark the positions of variants to allow for patterns amongst different VCFs to be clearly visible. We also plan to normalize variants so that different representations of the same underlying variation are not treated as distinct. Furthermore, we plan on providing a variant comparison methodology which is capable of assessing calls based on their similarity to expected results. A contemporary tool that provides this functionality on the command line is vcfdist [16].

Implementing a dedicated screen for users to directly add metadata information through the web interface would improve user experience and data organization. Furthermore, providing a metadata extraction option that leverages VCF file headers and filenames to deduce certain aspects of metadata would reduce manual input efforts. Providing long-term storage of user data and analyses by implementing user accounts would be helpful for users to compare their past analyses with one another as well as to rerun them with different options.

Commonly used golden sets could be made available by the server directly. The option to use precompiled high performance software tools for VCF file filtering could be provided to reduce processing times. Lastly, preserving VCF file annotations and allowing their use within the application for filtering and analysis would allow for greater flexibility in VCF Observer’s usage.

## Conclusions

This paper introduces VCF Observer, a novel software tool for analyzing, comparing, and visualizing VCF files. VCF Observer is a web tool with a user-friendly graphical interface that offers commonly performed functionality. It aims to aid in the preliminary analysis of the rapidly growing volume of genomic data produced as a result of advances in NGS. There are currently no graphical software tools for comparing or benchmarking VCF files, as well as many other common operations. VCF Observer addresses this issue by providing a graphical user interface through which many common operations including comparison, benchmarking, filtering (PASS filter and stringency), grouping (based on file metadata), and visualization (Venn diagrams, clustergrams, and precision–recall plots) can be performed. VCF Observer provides an intuitive interface for researchers and clinicians to gain a high-level understanding of variant data without needing any programming knowledge, enhancing the accessibility of bioinformatics.

## Data Availability

Project name: VCF Observer, Project home page: https://github.com/MBaysanLab/vcf-observer, Operating system(s): Platform independent, Programming language: Python 3, Other requirements: None, License: MIT, Any restrictions to use by non-academics: None, Sequencing data analyzed as part of this study is available at https://ftp.ncbi.nlm.nih.gov/ReferenceSamples/seqc and https://zenodo.org/records/5275189.

## Abbreviations

VCF: Variant call format
BCF: Binary VCF
GUI: Graphical user interface
MVC: Model–view–controller
CSV: Comma separated values
BED: Browser extensible data
Navbar: Navigation bar
WGS: Whole genome sequencing
WES: Whole exome sequencing
